# Pomegranate Bioactive Constituents Suppress Cell Proliferation and Induce Apoptosis in an Experimental Model of Hepatocellular Carcinoma: Role of Wnt/**β**-Catenin Signaling Pathway

**DOI:** 10.1155/2013/371813

**Published:** 2013-03-28

**Authors:** Deepak Bhatia, Roslin J. Thoppil, Animesh Mandal, Karishma A. Samtani, Altaf S. Darvesh, Anupam Bishayee

**Affiliations:** ^1^Cancer Therapeutics and Chemoprevention Group, Department of Pharmaceutical Sciences, College of Pharmacy, Northeast Ohio Medical University, Rootstown, OH 44272, USA; ^2^Department of Pharmaceutical Sciences, School of Pharmacy, American University of Health Sciences, Signal Hill, CA 90755, USA

## Abstract

Hepatocellular carcinoma (HCC) is the third leading cause of cancer-related death worldwide, and chemoprevention represents a viable approach in lowering the mortality of this disease. Pomegranate fruit, an abundant source of anti-inflammatory phytochemicals, is gaining tremendous attention for its wide-spectrum health benefits. We previously reported that a characterized pomegranate emulsion (PE) prevents diethylnitrosamine (DENA)-induced rat hepatocarcinogenesis though inhibition of nuclear factor-kappaB (NF-**κ**B). Since NF-**κ**B concurrently induces Wnt/**β**-catenin signaling implicated in cell proliferation, cell survival, and apoptosis evasion, we examined antiproliferative, apoptosis-inducing and Wnt/**β**-catenin signaling-modulatory mechanisms of PE during DENA rat hepatocarcinogenesis. PE (1 or 10 g/kg) was administered 4 weeks before and 18 weeks following DENA exposure. There was a significant increase in hepatic proliferation (proliferating cell nuclear antigen) and alteration in cell cycle progression (cyclin D1) due to DENA treatment, and PE dose dependently reversed these effects. PE substantially induced apoptosis by upregulating proapoptotic protein Bax and downregulating antiapoptotic protein Bcl-2. PE dose dependently reduced hepatic **β**-catenin and augmented glycogen synthase kinase-3**β** expression. Our study provides evidence that pomegranate phytochemicals exert chemoprevention of hepatic cancer through antiproliferative and proapoptotic mechanisms by modulating Wnt/**β**-catenin signaling. PE, thus, targets two interconnected molecular circuits (canonical NF-**κ**B and Wnt/**β**-catenin pathways) to exert chemoprevention of HCC.

## 1. Introduction

Hepatocellular carcinoma (HCC), the major primary malignant tumor of the liver, is one of the most life-threatening human cancers in the world, resulting in almost one million deaths every year [1]. There has been a drastic surge (70% increase) in the incidence of HCC in the United States during the last quarter century with nearly 29,000 new cases and more than 20,000 deaths expected to occur in 2012 alone [2]. Lack of effective diagnostic tools for early detection and limited treatment options for patients with advanced HCC contribute to a dismal prognosis coupled with high mortality for this disease. A critical need exists for the discovery and development of novel preventive as well as therapeutic strategies to combat the current morbidity and mortality associated with HCC [3–6]. 

Persistent oxidative stress and unresolved inflammation are two primary driving forces behind the development and exacerbation of HCC [7–9]. Lingering infection with hepatitis B virus (HBV) and hepatitis C virus (HCV) represents the major risk factor for HCC [10]. Chronic liver disease has been proposed to be a predisposing factor for at least 80% of HCC cases [11]. As a matter of fact, any condition that is linked to development of fibrosis and cirrhosis is strongly associated with the occurrence of HCC. In response to hepatocyte injury due to various factors, including inflammation and oxidative stress, hepatic stellate cells and portal fibroblasts undergo activation and transformation, resulting in fibrosis and ultimately cirrhosis [12]. Under these circumstances, the surviving hepatocytes proliferate to regenerate the injured liver. This cellular proliferation in the background of sustained inflammation and oxidative stress embodies a driving force for hepatic tumorigenesis [13]. Identification of cellular pathways necessary for the proliferation and survival of malignant cells in HCC not only aids in understanding the pathophysiology, diagnosis, and progression, but also provides a valuable tool in designing effective prevention and intervention of the disease. One such relevant pathway is the Wnt/*β*-catenin signaling cascade which plays a decisive role in cell fate, favoring cell proliferation over apoptosis. Accumulating evidence has shown that the canonical Wnt/*β*-catenin pathway is frequently activated in HCC and responsible for initiation and progression of the disease [13–16], and this pathway could be an attractive and viable target for chemoprevention and therapy of HCC [17–19].

The pomegranate (*Punica granatum*, Punicaceae), a unique, mystical, and distinctive fruit, is a native of the Himalayas in India and extensively cultivated in India, Israel, Spain, and United States. In addition to its ancient historical uses, pomegranate is used in several systems of medicine for a wide variety of ailments [20, 21]. The *“superfruit”* pomegranate is receiving substantial importance because of its powerful antioxidant and anti-inflammatory properties attributed to polyphenolic components (such as anthocyanins), hydrolysable tannins (e.g., ellagitannins and gallotannins), and condensed tannins (proanthocyanidins) [22–26]. Pomegranate constituents have been shown to possess exceptional health effects, such as protection against and/or treatment of cancer, neurodegenerative diseases, inflammation, ulcers, diabetes, dental ailments, high cholesterol, cardiovascular disease, obesity, bacterial infections, erectile dysfunction, and male infertility (reviewed in [27, 28]). Pomegranate extracts and purified phytochemicals suppress the proliferation of human breast, prostate, lung, and colon cancer cells *in vitro *as well as prevent and/or treat breast, skin, lung, colon, and prostate tumors in preclinical animal models (reviewed in [24, 29–31]). Several phase II clinical trials have linked oral consumption of pomegranate juice with significant prolongation of prostate-specific antigen (PSA) doubling time for men with prostate carcinoma with no accompanying serious adverse effects [32, 33]. 

Although pomegranate products have shown promising antitumor activities in various organs of animals, the chemopreventive potential of pomegranate has not been investigated against the preclinical model of hepatic tumorigenesis until very recently. Our laboratory has provided evidence, for the first time, that pomegranate-derived phytoconstituents exert a significant chemopreventive efficacy against dietary hepatocarcinogen diethylnitrosamine (DENA)-induced liver tumorigenesis in rats by potent antioxidant mechanisms mediated by upregulation of hepatic antioxidant and phase 2 genes [34]. Very recently, we have shown that suppression of the inflammatory cascade through modulation of nuclear factor-kappaB (NF-*κ*B) signaling pathway represents a novel mechanism of liver tumor inhibitory effects of pomegranate phytochemicals against experimental hepatocarcinogenesis [35]. It is well known that NF-*κ*B, the cardinal regulator of inflammation, acts as a hub for a number of interconnected signaling pathways implicated in malignancy, such as cell proliferation, cell survival, differentiation, apoptosis, invasion, angiogenesis, and metastasis [36]. Emerging evidence strongly suggests that NF-*κ*B may exhibit oncogenic potential by coordinately activating the canonical Wnt/*β*-catenin signaling pathway [37–39]. Functional proteomics analysis revealed that enhanced expression of Wnt-1 protein associated with NF-*κ*B might be an important mechanism of hepatitis B- and C-related HCC [40]. Based on multiple targets of NF-*κ*B and the possibility of crosstalk between NF-*κ*B and Wnt/*β*-catenin signaling pathways as well as previous reports from this laboratory [34, 35], we have hypothesized that (i) pomegranate-mediated chemoprevention of experimental hepatocarcinogenesis could be achieved by inhibition of abnormal hepatocyte proliferation and promotion of apoptosis; and (ii) antiproliferative effects pomegranate phytochemicals may be linked to suppression of activated Wnt/*β*-catenin signaling. The current study was therefore initiated to investigate the extent of cell proliferation and apoptosis during DENA-induced chemical rat liver carcinogenesis in the presence or absence of pomegranate treatment. The involvement of Wnt/*β*-catenin signaling under the same experimental condition has also been examined by monitoring the expression of several key components of this pathway as a possible mechanism of pomegranate chemoprevention of experimental hepatic malignancy. 

## 2. Materials and Methods

### 2.1. Materials

Pomegranate emulsion (PE) was purchased from Rimonest Ltd., Haifa, Israel. We previously provided the detailed description of the preparation of this formulation [34]. The chemical analyses of this product showed the presence of mixed octadecatrienoic acids, sterols and steroids (such as 17-*α*-estradiol), tocol, *γ*-tocopherol in the lipid phase and caffeic acid, corilagin, ellagic acid, ferulic acid, gallic acid, 5-hydroxymethylfurfural, protocatechuic acid, punicalagins (A and B), and *trans*-*p*-coumaric acid in the aqueous phase [34]. Paraformaldehyde was procured from Ted Pella Inc., Redding, CA. Primary antibodies, such as mouse monoclonal proliferating cell nuclear antigen (PCNA, sc-56), rabbit polyclonal cyclin D1 (sc-753), mouse monoclonal Bax (sc-70407), mouse monoclonal Bcl-2 (sc-7382), rabbit polyclonal *β*-catenin (sc-7199), rabbit polyclonal glycogen synthase kinase-3*β* (GSK-3*β*, sc-9166), *β*-actin (sc-47778), and mouse and rabbit ABC staining systems were purchased from Santa Cruz Biotechnology (Santa Cruz, CA). TdT-FragEL DNA fragmentation detection assay kit was procured from EMD Biosciences, Inc. (San Diego, CA). Pierce BCA protein assay kit was obtained from Thermo Scientific (Rockford, IL). Quick RNA mini Prep kit and Verso cDNA synthesis kit were purchased from Zymo Research Corporation (Irvine, CA) and Thermo Scientific (Waltham, MA), respectively. 

### 2.2. In Vivo Experimental Protocol and Tissue Harvesting

Liver tissue used for all assays in this study was harvested from our previously published chemopreventive study in which male Sprague-Dawley rats (Harlan Laboratories, Indianapolis, IN) fed with oral PE at a dose of 1 or 10 g/kg body weight exhibited 26 or 50% inhibition of hepatic nodule incidence, respectively [34]. The animal experimentation was carried out at the Northeast Ohio Medical University (Rootstown, OH) following the animal protocol approved by the Institutional Animal Care and Use Committee. In brief, following an acclimatization period (7 days), the animals were randomly divided into five groups. While group A animals were maintained as untreated normal control, group B animals were administered with a sham emulsion (Rimonest Ltd., Haifa, Israel) via oral gavage at a dose of 10 g/kg three times/week (Monday, Wednesday, and Friday). Three other animal groups (groups C, D, and E) were similarly fed with PE at 1 g/kg (groups C) or 10 g/kg (groups D, and E). The aforementioned oral feeding was continued for 4 weeks, and then hepatocarcinogenesis was initiated in animals from groups B, C and D by single intraperitoneal (*i.p.*) injection of DENA (200 mg/kg). Following a recovery period of two weeks, phenobarbital (PB, a well-known promoter of rat liver carcinogenesis) was introduced in the drinking water of DENA-exposed animals at a concentration of 0.05% (w/v). Oral feeding of rats with PE or sham emulsion was continued till the end of the animal experiment. All animals were sacrificed 18 weeks following the DENA administration, that is, 22 weeks after commencement of the study. Liver tissues from various rat groups were collected and either preserved in paraformaldehyde for immunohistochemical work or immediately snap frozen in liquid nitrogen, stored at –70°C, and used for molecular studies as described in the following. 

### 2.3. Immunohistochemical Determination

Serial sections of liver tissue were prepared using a cryostat (Leica Microsystems, Nussloch, Germany) and used for immunohistochemical analysis of PCNA, cyclin D1, Bax, Bcl-2, *β*-catenin, and GSK-3*β* protein expressions following our published methods [41]. The presence of apoptotic cells in liver sections was detected by TdT-FragEL DNA fragmentation detection assay kit following the manufacturer's protocol, as we described earlier [42]. The immunohistochemical slides were visualized under a light microscope, and 1,000 hepatocytes/animal were analyzed. The labeling index (LI) and apoptotic index (AI) were expressed as the number of PCNA-positive and apoptotic cells per 100 hepatocytes, respectively. All other immunohistochemical results were expressed as percentage of immunopositive cells. 

### 2.4. Western Blot Analysis

Frozen liver tissue samples were homogenized in ice-cold RIPA lysis buffer (containing 50 mM Tris-HCl, 1% Nonidet P-40, 0.5% sodium deoxycholate, 40 mM NaF, 10 mM NaCl, 10 mM Na_3_VO_4_, 1 mM phenylmethanesulfonyl fluoride, and 10 mM dithiothreitol) to produce a 10% w/v tissue homogenate. The sample was then centrifuged at 4°C at 14,000 ×g for 20 min. The supernatant was collected, and protein concentration was estimated using the Pierce BCA protein assay kit following the accompanying instructions. Equal amounts of protein samples were loaded and run on a 10% Tris-HCl gel (Bio-Rad Laboratories, Hercules, CA), transferred onto a nitrocellulose membrane, and separately probed with anti-cyclin D1, anti-*β*-catenin, anti-GSK-3*β*, or anti-*β*-actin (all 1 : 1000 dilution) antibody. The transferred proteins were visualized by an enhanced chemiluminescence detection system (Thermo Scientific, Rockford, IL) and analyzed using a Kodak analyzer. Loading of equal amounts of protein was ensured by *β*-actin.

### 2.5. Gene Expression Studies

 Total RNA from ~20 mg of liver sample was extracted using Quick RNA mini Prep kit as per the vendor's protocol. Complementary DNA (cDNA) was synthesized from 1 *μ*g of total RNA using the Verso cDNA synthesis kit following the manufacturer's instructions. Polymerase chain reaction (PCR) was performed using specific primers for rat cyclin D1, *β*-catenin, GSK-3*β*, and glyceraldehyde 3-phosphate dehydrogenase (GAPDH) using following primer pairs: cyclin D1 sense: 5′-GCGTACCCTGACACCAATCT-3′; antisense: 5′-GGCTCCAGAGACAAGAAACG; *β*-catenin sense: 5′-GCCAGTGGATTCCGTACTGT-3′; antisense: 5′-GAGCTTGCTTTCCTGATTGC; GSK-3*β* sense: 5′-CAAGCAGACACTCCCTGTGA-3′; antisense: 5′-GTGGCTCCAAAGATCAGCTC; GAPDH sense: 5′-AGACAGCCGCATCTTCTTGT-3′; and antisense: 5′-TACTCAGCACCAGCATCACC-3′. These primer sequences were designed utilizing the Primer3 program and synthesized by Eurofins MWG Operon (Huntsville, AL). The PCR products were analyzed by agarose gel electrophoresis and visualized using ethidium bromide staining. GAPDH was used as the housekeeping gene.

### 2.6. Statistical Analysis

All quantitative data reported here are presented as mean ± standard error of mean (SEM). Significant differences among various animal groups were detected by one-way ANOVA with *post hoc* analysis performed by the Student-Neuman-Keuls test. A *P* value less than 0.05 was considered statistically significant. Statistical analysis and graphical representation of data were performed using SigmaStat 3.1 software (Systat software, Inc., San Jose, CA).

## 3. Results 

### 3.1. Antiproliferative Cellular Mechanism of Pomegranate in DENA-Induced Hepatocarcinogenesis

**Figure 1:**
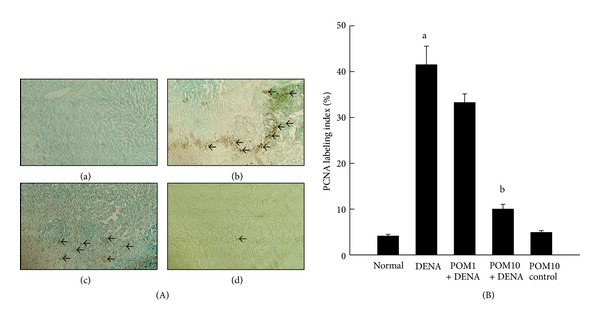
Effects of PE on PCNA expression during DENA-initiated hepatocarcinogenesis in rats. (A) Representative immunohistochemical localization of PCNA (magnification: 100x). Arrows indicate immunohistochemical staining of PCNA. Several groups are (a) normal control; (b) DENA control; (c) PE 1 g/kg plus DENA; and (d) PE 10 g/kg plus DENA. (B) Quantification of PCNA-positive cells based on 1,000 hepatocytes per animal and 4 animals per group. Each bar represents the mean ± SEM (*n* = 4).  ^a^ *P* < 0.001 as compared with normal group;  ^b^ *P* < 0.001 as compared with DENA control.

As shown in (Figure 1(A)a–d), the immunohistochemical staining depicts the differential expression levels of the proliferative marker, PCNA, in liver sections obtained from the various experimental rat groups. Near to complete absence of PCNA-positive cells was observed in both normal control (Figure 1(A)a) and only PE-treated (10 g/kg) (figure not shown) groups. An increase in hepatic PCNA expression was contrastingly observed in animals treated with DENA alone (Figure 1(A)b), while PE treatment (1 g/kg) in conjunction with DENA exposure resulted in insignificant decreases in PCNA-positive expression (Figure 1(A)c). These elevated PCNA levels were shown to be subsequently reversed in the sections obtained from rats treated with PE at 10 g/kg in addition to DENA treatment (Figure 1(A)d). Figure 1(B) represents the quantitative analysis performed on the immunohistochemical data, which shows significant (*P* < 0.001) elevation in the frequency of PCNA expressing hepatocytes (PCNA LI) in the DENA-treated animals comparing to the normal control. In comparison to the DENA-control rats, PE treatment is shown to decrease the number of PCNA-positive cells. Nevertheless, a significant (*P* < 0.001) reduction was only achieved in rats treated with PE at the highest dose of 10 g/kg.

### 3.2. Differential Expression of Cell Cycle Specific Gene—Cyclin D1—in the Presence and Absence of Pomegranate Treatment in Hepatocarcinogenesis

**Figure 2:**
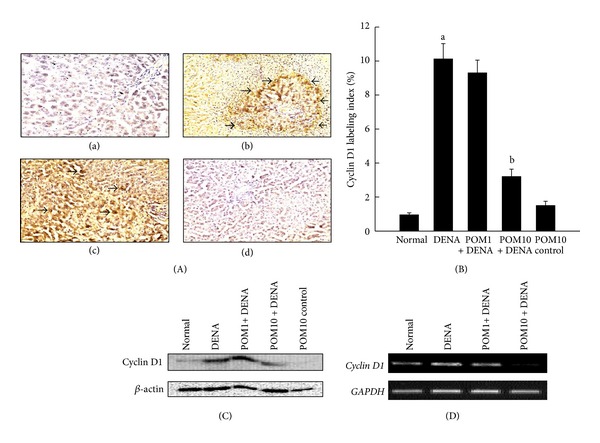
Effects of PE on cyclin D1 expression during DENA-mediated hepatocarcinogenesis in rats. (A) Representative immunohistochemical localization of cyclin D1 (magnification: 100x). Arrows indicate immunohistochemical staining of cyclin D1. Several groups are (a) normal control; (b) DENA control; (c) PE 1 g/kg plus DENA; and (d) PE 10 g/kg plus DENA. (B) Quantification of cyclin D1-positive cells based on 1,000 hepatocytes per animal and 4 animals per group. Each bar represents the mean ± SEM (*n* = 4).  ^a^ *P* < 0.001 as compared with normal group;  ^b^ *P* < 0.001 as compared with DENA control. (C) Representative Western blot indicating protein expression levels of cyclin D1 in various experimental groups and (D) representative RT-PCR analysis of cyclin D1 expression in various groups of rats. Total hepatic RNA was isolated, subjected to reverse transcription, and resulting cDNA was subjected to RT-PCR analysis using specific primer sequence. The *GAPDH* was used as the housekeeping gene.

Represented in Figure 2(A)a–d is the immunocytochemical data depicting the expression of cell cycle specific gene cyclin D1 in hepatic sections procured from several experimental animals. Cyclin D1 was found to be highly expressed in the DENA-control rats (Figure 2(A)b), whereas this observation was not made in the rat liver sections obtained from the normal control (Figure 2(A)a) as well as PE control (figure not shown) groups. A drastic reduction in the numbers of cyclin D1 immunoreactive cells was recorded in rats treated with PE at 10 g/kg (Figure 2(A)d). The subsequent quantitative analysis performed denotes a significant (*P* < 0.001) increase in the percentage of cyclin D1-positive cells in the rats administered with DENA with respect to the normal control rats (Figure 2(B)). Elevated immunohistochemical expression of cyclin D1 was significantly (*P* < 0.001) abrogated only in the rats treated with PE, at the highest dose of 10 g/kg, when compared to the DENA-challenged rats, although reduction in the percentage of immunopositive cells was noted in all PE-treated groups. These data are further confirmed utilizing Western blotting technique and RT-PCR (Figures 2(C) and 2(D)). Hepatic cyclin D1 mRNA and protein expression levels were clearly upregulated in the DENA-treated animals, while PE treatment at both doses was found to consistently decrease transcriptional and translational levels of cyclin D1.

### 3.3. Proapoptotic Potential of Pomegranate in DENA-Evoked Hepatocarcinogenesis

**Figure 3:**
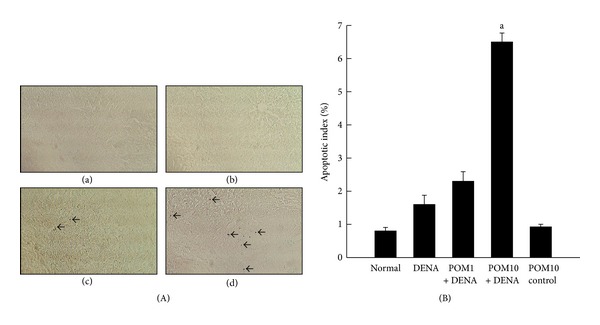
Effects of PE on apoptosis in DENA-initiated hepatocarcinogenesis in rats. (A) Representative immunohistochemical localization of apoptotic cells (magnification: 100x). Arrows indicate immunohistochemical staining of apoptotic cells. Several groups are (a) normal control; (b) DENA control; (c) PE 1 g/kg plus DENA; and (d) PE 10 g/kg plus DENA. (B) Quantification of immunopositive cells based on 1,000 hepatocytes per animal and 4 animals per group. Each bar represents the mean ± SEM (*n* = 4).  ^a^ *P* < 0.001 as compared with DENA-control group.

The DNA fragmentation assay performed on the rat liver sections indicated almost no apoptotic cells in both the normal control group (Figure 3(A)a) and PE-administered group (figure not shown). Animals exposed to DENA alone (Figure 3(A)b) were similarly found to have absolutely no immunopositive cells which, upon treatment with PE at both doses, was found to be increased (Figure 3(A)c-d). Interestingly, a substantial increase in apoptotic cells was noticed in liver sections obtained from animals treated with PE at 10 g/kg (Figure 3(A)d). Quantitative analysis of immunohistochemical data revealed that the frequency of apoptotic cells were significantly (*P* < 0.001) elevated in the rats subjected to PE treatment at 10 g/kg in comparison to the animals administered with DENA alone (Figure 3(B)). 

### 3.4. Regulation of Apoptosis-Related Proteins by Pomegranate during Experimental Rat Liver Carcinogenesis

**Figure 4:**
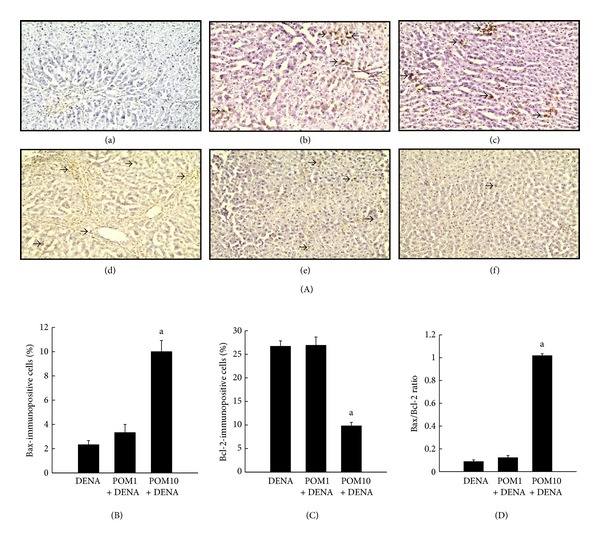
Effects of PE on Bax and Bcl-2 expressions during DENA-mediated hepatocarcinogenesis in rats. (A) Immunohistochemical detection of Bax and Bcl-2 expression in the presence or absence of PE. (a)–(c) Representative immunohistochemical localization of Bax (magnification: 100x). Arrows indicate immunohistochemical staining of Bax. (d)–(f) Representative immunohistochemical localization of Bcl-2 (magnification: 100x). Arrows indicate immunohistochemical staining of Bcl-2. Several groups are ((a) and (d)) DENA control; ((b) and (e)) PE 1 g/kg plus DENA; and ((c) and (f)) PE 10 g/kg plus DENA. (B) Quantification of Bax-positive cells based on 1,000 hepatocytes per animal, and 4 animals per group. Each bar represents the mean ± SEM (*n* = 4).  ^a^ *P* < 0.001 as compared with DENA control. (C) Quantification of Bcl-2-positive cells based on 1,000 hepatocytes per animal and 4 animals per group. Each bar represents the mean ± SEM (*n* = 4).  ^a^ *P* < 0.001 as compared with DENA control. (D) Quantification of Bax/Bcl-2 ratio of positive cells based on 1,000 hepatocytes per animal and 4 animals per group. Each bar represents the mean ± SEM (*n* = 4).  ^a^ *P* < 0.001 as compared with DENA control.

Liver sections, obtained from the different experimental rat groups, were immunostained for the proapoptotic protein Bax and the antiapoptotic protein Bcl-2, and the results are shown in Figure 4(A)a–f. Sections from rats exposed to DENA alone were shown to have very limited expression of the proapoptotic protein Bax (Figure 4(A)a), while treatment with PE at 1 g/kg (Figure 4(A)b) and 10 g/kg (Figure 4(A)c) reflected an increase in the expression of Bax. In stark contrast, immunostaining for the antiapoptotic protein, Bcl-2, in the DENA-control liver sections yielded very high expression levels of the protein (Figure 4(A)d) which was subsequently reduced with PE exposure at both doses (Figure 4(A)e-f). Figures 4(B) and 4(C) represent the affirmative corresponding quantitative analysis of the immunohistochemical data associated with Bax and Bcl-2, respectively. Although an increase in the proapoptotic protein Bax is seen in the PE-treated (1 g/kg) rats, significant (*P* < 0.001) increases in the percentage of Bax-positive cells were clearly observed in the animals exposed to the highest dose of PE (10 g/kg), when compared to the DENA-exposed rats (Figure 4(B)). Figure 4(C) indicates a very high expression of the antiapoptotic protein Bcl-2 in the hepatocytes of DENA-control rats as well as in those of PE-treated (1 g/kg) animals; however, treatment with PE at the highest dose (10 g/kg) significantly (*P* < 0.001) decreased the increased expression of Bcl-2. A significant (*P* < 0.001) elevation in the Bax/Bcl-2 ratio was observed in the liver tissue harvested from the PE-treated (10 g/kg) rats in comparison to the DENA-exposed animals (Figure 4(D)).

### 3.5. Reversal of DENA-Mediated Induction of Hepatic *β*-Catenin Expression by Pomegranate

**Figure 5:**
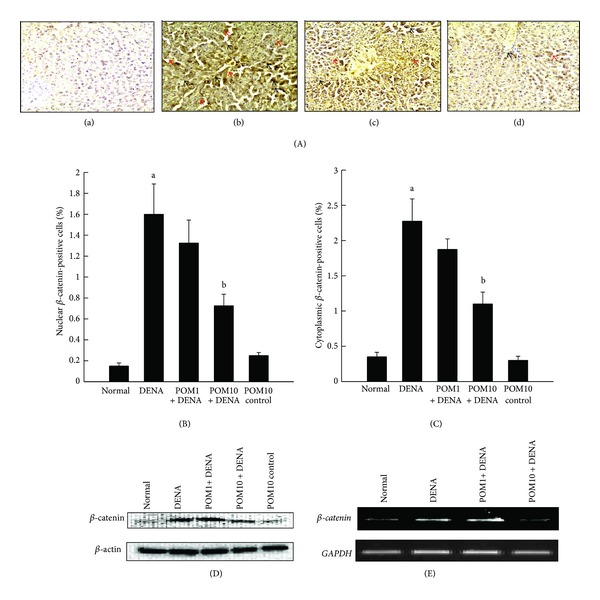
Effects of PE on *β*-catenin expression during DENA-evoked hepatic preneoplasia in rats. (A) Representative immunohistochemical localization of *β*-catenin in nucleus ((b), (c), and (d); red arrows) and cytosol ((b), (c), and (d); black arrows) (magnification: 100x). Several groups are (a) normal control; (b) DENA control; (c) PE 1 g/kg plus DENA; and (d) PE 10 g/kg plus DENA. (B) Quantification of nuclear *β*-catenin-immunopositive cells in rat livers of several experimental groups. One thousand hepatocytes were counted per animal and the results were based on 4 animals per group. Each bar represents the mean ± SEM (*n* = 4).  ^a^ *P* < 0.001 as compared to normal group;  ^b^ *P* < 0.05 as compared to DENA control. (C) Quantification of cytoplasmic *β*-catenin-immunopositive cells in rat livers of several experimental groups. One thousand hepatocytes were counted per animal, and the results were based on 4 animals per group. Each bar represents the mean ± SEM (*n* = 4).  ^a^ *P* < 0.001 as compared to normal group;   ^b^ *P* < 0.05 as compared to DENA control. (D) Representative Western blot indicating protein expression levels of *β*-catenin in various experimental groups and (E) representative RT-PCR analysis of *β*-catenin expression in various groups of rats. Total hepatic RNA was isolated, subjected to reverse transcription, and resulting cDNA was subjected to RT-PCR analysis using specific primer sequence. The *GAPDH* was used as the housekeeping gene.

Based on immunohistochemical analysis,variations in the nuclear and cytosolic expressions of *β*-catenin were clearly observed in the liver sections harvested from several groups of animals (Figure 5(A)a–d). Expression of immunopositive cells was clearly absent in sections obtained from both the normal control (Figure 5(A)a) as well rats treated with only PE (figure not shown). Highly elevated frequency of both nuclear and cytosolic *β*-catenin-positive cells was recorded in rats exposed to DENA (Figure 5(A)b). In comparison, the rats treated with different doses of PE (1 g/kg and 10 g/kg) had decreased expression of nuclear as well as cytosolic expression of *β*-catenin (Figures 5(A)c and 5(A)d). Interestingly, the decrease was more pronounced in the animals treated with the higher dose of PE. The corresponding quantitative analysis presented in Figures 5(B) and 5(C) confirms our immunostaining data, indicating a significant (*P* < 0.001) increase in nuclear and cytosolic *β*-catenin expression in DENA-control rats, compared to the normal control, respectively. Treatment with PE at 1 g/kg did not significantly reduce the elevated levels of *β*-catenin at both the nuclear and cytosolic front; however, PE treatment at 10 g/kg significantly (*P* < 0.05) decreased both nuclear and cytosolic *β*-catenin expression. Further confirmation of these results was shown utilizing Western blotting method as well as RT-PCR technique (Figures 5(D) and 5(E), resp.). The former shows elevated protein levels of *β*-catenin in the DENA-administered rats in comparison to the normal control rat livers, while PE administration reversed this condition and brought down the high expression of *β*-catenin. Liver samples from the different experimental groups, subjected to RT-PCR analysis, depicted a high mRNA expression level of *β*-catenin in the DENA-control rat livers, while markedly decreased expression was witnessed with PE treatment, especially at the highest dose of 10 g/kg. Interestingly, the basal transcriptional (data not shown) and translational (Figure 5(D)) levels of *β*-catenin were unaltered by the highest dose of PE.

### 3.6. Pomegranate-Mediated Modulation of Hepatic GSK-3*β* Expression in Experimental Rat Liver Carcinogenesis

**Figure 6:**
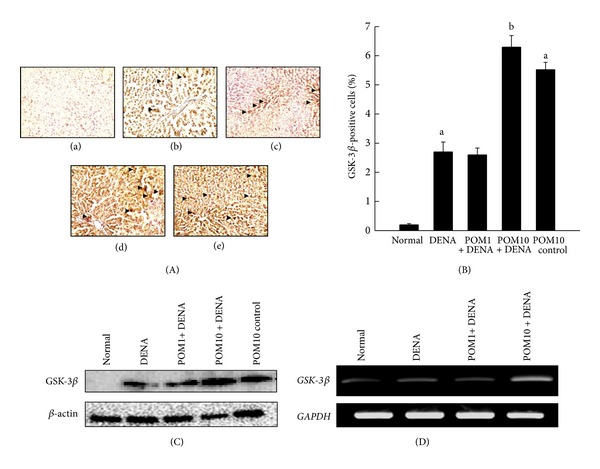
Effects of PE on GSK-3*β* expressions during DENA-evoked hepatic preneoplasia in rats. (A) Representative immunohistochemical localization of GSK-3*β* (magnification: 100x) in different rat groups. Several groups are (a) normal control; (b) DENA control; (c) PE 1 g/kg plus DENA; (d) PE 10 g/kg plus DENA; and (e) PE 10 g/kg control. (B) Quantification of hepatic GSK-3*β*-immunopositive cells. One thousand hepatocytes were counted per animal, and the results were based on 4 animals per group. Each bar represents the mean ± SEM (*n* = 4).  ^a^ *P* < 0.001 as compared to normal group;  ^b^ *P* < 0.001 as compared to DENA control. (C) Representative Western blot indicating protein expression levels of GSK-3*β* in various experimental groups and (D) representative RT-PCR analysis of GSK-3*β* expression in various groups of rats. Total hepatic RNA was isolated, subjected to reverse transcription, and resulting cDNA was subjected to RT-PCR analysis using specific primer sequence. The *GAPDH* was used as the housekeeping gene.

Represented in Figure 6(A)a–e is the immunohistochemical staining of GSK-3*β* in rat liver sections from the various experimental groups. The normal control group demonstrated very low expression of GSK-3*β* (Figure 6(A)a). A moderate increase in the expression of GSK-3*β* was observed in the DENA-control group (Figure 6(A)b), while treatment with PE at 1 g/kg did not alter the expression of GSK-3*β* (Figure 6(A)c). However, treatment with PE at 10 g/kg depicted a substantial elevation in the number of immunopositive cells for GSK-3*β* (Figure 6(A)d). An elevated expression of GSK-3*β* was also observed in the PE-control liver sections (Figure 6(A)e) compared to normal control as well. The corresponding immunohistochemical analysis of percentage of immunopositive cells is represented in Figure 6(B). The quantitative expression of GSK-3*β* in the DENA-control group was significantly (*P* < 0.001) greater compared to that observed in the normal group. Treatment with PE at 10 g/kg significantly (*P* < 0.001) elevated the numbers of GSK-3*β*-immunopositive cells compared to DENA-control, whilst PE treatment at the lower dose did not change expression levels. Interestingly, a significant (*P* < 0.001) increase in GSK-3*β* levels was noticed in PE-control animals in comparison to the normal control. Our immunohistochemistry data provide support to the Western blot analysis of GSK-3*β* protein levels in liver samples collected from the experimental animals (Figure 6(C)). The protein level of GSK-3*β* was slightly increased in DENA-administered animals compared to the normal, while treatment with PE at both doses as well as PE control resulted in upregulated protein expressions of GSK-3*β*. The mRNA analysis of the liver samples harvested from the experimental rats was performed by the technique of RT-PCR, and results are depicted in Figure 6(D). The data are in affirmative of our immunohistochemical and Western data, conclusively exhibiting slightly elevated gene expression levels of GSK-3*β* in DENA-exposed animals than in normal-control animals. PE treatment at 10 g/kg was noted to have elevated mRNA expression of GSK-3*β* comparing to DENA-control animals. A conspicuous upregulation of GSK-3*β* expression at both protein (Figure 6(C)) and mRNA (data not shown) was visible following treatment with the highest dose of PE compared to normal control. 

## 4. Discussion

We have recently shown that a complex pomegranate product (PE) containing a wide spectrum of phytoconstituents afforded a striking chemoprevention against chemically induced hepatocarcinogenesis in rats [34]. It is plausible that various phytochemicals present in PE may target several interconnected molecular pathways to inhibit liver tumorigenesis. Supporting this possibility, we have provided evidence that PE simultaneously target Nrf2-regulated redox signaling and NF-*κ*B-mediated inflammatory pathway to achieve chemoprevention of experimental hepatocarcinogenesis [34, 35]. In the present study, we set ourselves at the aim of investigating additional and related mechanisms as regards to our previous findings to provide a comprehensive understanding of pleiotropic mechanisms of action of pomegranate phytochemicals in preventing liver cancer. 

Cell proliferation plays a fundamental role in the multistep carcinogenesis process, including initiation, promotion, and progression, and is considered to be an important mechanism for the pathogenesis of HCC [43]. Hence, exploration of agents that can affect abnormal proliferation of hepatocytes may be of immense value in the prevention of HCC. PCNA functions as a cofactor of DNA polymerase and thus is directly involved in DNA replication. The positive expression of PCNA is considered a common index for hepatocyte proliferation at late G1- and early S-phase. PCNA represents an important cellular marker for assessing the proliferation during hepatocarcinogenesis [44]. In our study, the expression of PCNA was examined immunohistochemically in livers from several animal groups. The elevated expression of PCNA, resulting in a drastic increase in PCNA LI, in the liver sections of DENA-treated animals indicates accelerated cell proliferation during an early phase of rat liver tumorigenesis, which supports our previous observations [41, 42]. Significant decreases in the number of PCNA-positive hepatocytes and consequently PCNA LI due to PE treatment clearly indicate a decrease in hepatocyte proliferation, possibly through inhibition of DNA synthesis during experimental hepatocarcinogenesis. It is mostly likely that the antiproliferative mechanisms of pomegranate phytochemicals eventually contributed to inhibition of DENA-induced tumorigenesis, as we previously observed [34]. Our data are in agreement of several studies reported from other laboratories showing *in vitro* and *in vivo* antiproliferative effects of various pomegranate phytochemicals in several cancer models [24, 45].

Cyclin D1, a cell cycle regulatory protein, is responsible for the transition from G1- to S-phase [46]. As cyclin D1 possesses a critical role for cell cycle progression, dysregulated expression of cyclin D1 gene may contribute to cellular genomic instability and malignancy [47]. Elevated expressions of cyclin D1 gene and protein disrupting G1/S regulatory point of the cell cycle have been found to lead abnormal cell proliferation during DENA-induced sequential hepatocarcinogenesis in rats [48–50]. As increased expression of cyclin D1 protein has been found in HCC patients [51] and correlated with poor overall survival in HCC patients [52], it can be used as a prognostic marker and therapeutic target. The results of our immunohistochemical as well as Western blot analysis clearly demonstrate an upregulation of cyclin D1 protein expression in the livers isolated from DENA control animals. These findings are supported by the increased expression of cyclin D1 gene following DENA treatment. Collectively, our data underscore the critical role of cell cycle regulatory protein in DENA hepatocellular carcinogenesis in rats under our experimental conditions. Interestingly, a similar transcriptional or translational upregulation of cyclin D1 in response to DENA exposure in rodents has been reported by various other laboratories [53–55]. A near normal level of cyclin D1 expression was observed due to PE treatment. Reversal of dysregulation of a critical checkpoint of the cell cycle could be one of the possible mechanisms of pomegranate-mediated inhibition of heptotumorigenesis, and it underscores the value of targeting a cell cycle progression protein to achieve chemoprevention of hepatocellular cancer. 

Apoptosis or programmed cell death, a highly preserved cellular mechanism, is involved in tissue homeostasis through targeted elimination of singe cells without disrupting physiological function of a tissue. Apoptosis is marked by distinct morphological alterations characterized by cytoplasmic as well as nuclear condensation, DNA fragmentation, phosphatidylserine externalization, and plasma membrane blebbing [56]. Dysregulation of apoptosis disturbing the balance between cell proliferation and cell death contributes to development and progression of hepatic malignancy [57, 58]. Apoptosis detection in tumor mass has emerged as an important diagnostic tool and induction of tumor cell death by apoptosis has been accepted as one of the fundamental objectives of liver cancer therapy [59]. Promotion of apoptosis in tumor-target tissues has been identified as a novel mechanism of potential chemopreventive drug. In the present study, DNA fragmentation was detected to identify cells undergoing apoptosis. Our results show an extremely low order of DNA fragmentation in DENA-exposed rat livers indicating apoptosis evasion. PE treatment showed a drastic increase in the formation of DNA fragments which provides substantial evidence of induction of cell death by apoptosis and consequently reduction of proportion of transformed hepatocytes in DENA-treated animals. Our data showing apoptosis induction during experimental hepatocarcinogenesis in rats by pomegranate phytoconstituents are concordant with a large number of studies showing proapoptotic effects of pomegranate-derived agents in various *in vitro* and *in vivo* preclinical cancer models [reviewed in ref. [31]].

The apoptotic signal in hepatocytes is believed to be transmitted through the complex interaction of intercellular proteins. The transcription factor NF-*κ*B and members of the Bcl-2 family consisting of both proapoptotic and antiapoptotic proteins contribute to the regulation of apoptosis in hepatocytes [58]. Many of the genetic alterations of HCC lead to an imbalance in the proapoptotic and antiapoptotic proteins of the Bcl-2 family [60]. The antiapoptotic proteins, including Bcl-2 and Bcl-xL, are known to inhibit mitochondrial apoptotic pathway by blocking the release and oligomerization of proapoptotic proteins and are overexpressed in HCC [61]. On the other hand, proapoptotic members of the Bcl-2 family, such as Bax, Bad, and Bid, initiate mitochondrial apoptosis by facilitating pore formation and cytochrome *c* release from the inner mitochondrial membrane with subsequent activation of caspases resulting in cell death. These proapoptotic members are downregulated in HCC [62]. An alteration in Bax/Bcl-2 ratio plays a crucial role in deciding whether a cell should switch towards proliferation or apoptosis [63]. Our present study revealed that PE registered an increase in the expression of proapoptotic gene Bax and decrease in the level of antiapoptotic gene Bcl-2, resulting in an extraordinary surge in Bax/Bcl-2 ratio. Based upon these results, it is clear that pomegranate bioactive phytochemicals induce intrinsic apoptosis and facilitate elimination of transformed cells during hepatocellular carcinogenesis by targeting the Bcl-2 family members. 

The Wnt/*β*-catenin signaling pathway is implicated in liver physiology and pathology through regulation of various fundamental cellular events, including proliferation, differentiation, survival, inflammation, oxidative stress, morphogenesis, and regeneration [15, 64, 65]. In canonical Wnt pathway, the multifunctional protein *β*-catenin represents the key signaling intermediate. Under normal physiological conditions, free cytoplasmic *β*-catenin undergoes degradation through a coordinated action of a multiprotein destruction complex consists of GSK-3*β*, adenomatous polyposis coli, casein kinase 1*α* (CK1*α*), and Axin [66]. It is known that *β*-catenin is first phosphorylated at serine-45 by CK1*α*, facilitating its subsequent phosphorylation at serine-33, serine-37, and threonine-41 by GSK-3*β* [67, 68]. The phosphorylated *β*-catenin first undergoes ubiquitination by the cellular E3 ubiquitin ligase complex and then degradation by 26S proteasome [69]. In the event of Wnt ligands binding with the transmembrane Frizzled (FZD) receptor and coreceptor low-density lipoprotein receptor-related protein 5 or 6 (LRP 5/6), activation of the canonical Wnt pathway is initiated. In this process, FZD recruits cytoplasmic scaffolding protein Dishevelled (Dvl) which subsequently recruits Axin and GSK-3*β* to LRP 5/6 [70]. Consequently, LRP 5/6 is phosphorylated by CK1*α* and GSK-3*β*, resulting in disruption of the formation of the destruction complex and inhibition of GSK-3*β*-mediated phosphorylation of *β*-catenin. The net result is the stabilization and accumulation of *β*-catenin in the cytosol followed by its translocation to the nucleus. Once inside the nucleus, *β*-catenin interacts with transcription factor T-cell factor/lymphoid enhancer factor and eventually activates the transcription of various Wnt target genes implicated in a number of important biological functions, including proliferation (e.g., c-myc), cell cycle control (e.g., cyclin D1), apoptosis (e.g., survivin), and inflammation (e.g., COX-2) [71–74]. Aberrant constitutive activation of the Wnt/*β*-catenin pathway leads to uncontrolled cell proliferation, growth, and survival, promoting various human malignancies, including HCC [13, 16]. Emerging evidence based on experimental as well as clinical observations indicate the involvement of activated Wnt/*β*-catenin signaling at various stages of hepatic neoplasia, making it an attractive and viable target for prevention and treatment of HCC [reviewed in refs. [17–19]]. Accumulation of *β*-catenin in the cytoplasm and nucleus has previously been reported during stepwise progression of DENA-initiated hepatocarcinogenesis in rats [75] and DENA/PB-mediated HCC in mice [76]. Our immunohistochemical study also reveals that hepatocytes from DENA-control animals exhibit a marked overexpression of *β*-catenin in the cytoplasm and/or nucleus, confirming activation of Wnt/*β*-catenin signaling pathway at an early stage of DENA hepatocarcinogenesis in rats. The accompanying Western blot and RT-PCR data provide further support to our observation regarding induction of the canonical Wnt/*β*-catenin pathway. A dose-dependent inhibition of *β*-catenin expression in transcriptional and translational levels by pomegranate constituents clearly underscores the impairment of the canonical Wnt/*β*-catenin oncogenic signaling in rat liver carcinogenesis. 

Reduced expression of GSK-3*β* mRNA has been observed in human hepatic cancer linked to HCV infection [77]. HBV-X protein upregulated *β*-catenin with an inactivation of GSK-3*β* in liver cancer [78]. Targeted downregulation of *β*-catenin protein has been associated with increased GSK-3*β* protein expression in HCC [79]. A parallel induction of hepatic GSK-3*β* protein following pomegranate treatment has been an interesting finding of our study which indicates possible degradation of *β*-catenin protein via upregulation of GSK-3*β*. The activation of GSK-3*β* may be responsible, at least in part, for the attenuation of oncogenic Wnt/*β*-catenin signaling and its effect on downstream gene expression. While the involvement of GSK-3*β* in downregulation of *β*-catenin protein following pomegranate treatment is apparent from this study, it is not clear how pomegranate phytochemicals affect the expression of *β*-catenin at the transcriptional level and may suggest other mechanisms. Pomegranate bioactive compounds may have direct effects on transcriptional regulation of *β*-catenin and GSK-3*β* genes. This is in line with a recent study that shows reduction in mRNA expressions of *β*-catenin and GSK-3*β* in human hepatocarcinoma cells following treatment with nimbolide, a natural compound derived from neem tree [80]. Additionally, methylated chrysin, a constituent of pepper tree leaves, has been found to abrogate the elevated expression of *β*-catenin mRNA in preneoplastic lesions induced by DENA in rats [81]. Although emerging studies have provided credible evidence on the potential of a gamut of bioactive food components in the prevention and treatment of various neoplastic diseases by targeting the wingless signaling [reviewed in [82–84]], very limited information is available on chemoprevention of HCC by inhibiting activated Wnt/*β*-catenin signaling pathway using nutraceuticals and dietary components [reviewed in [19]]. 

## 5. Conclusion

**Figure 7:**
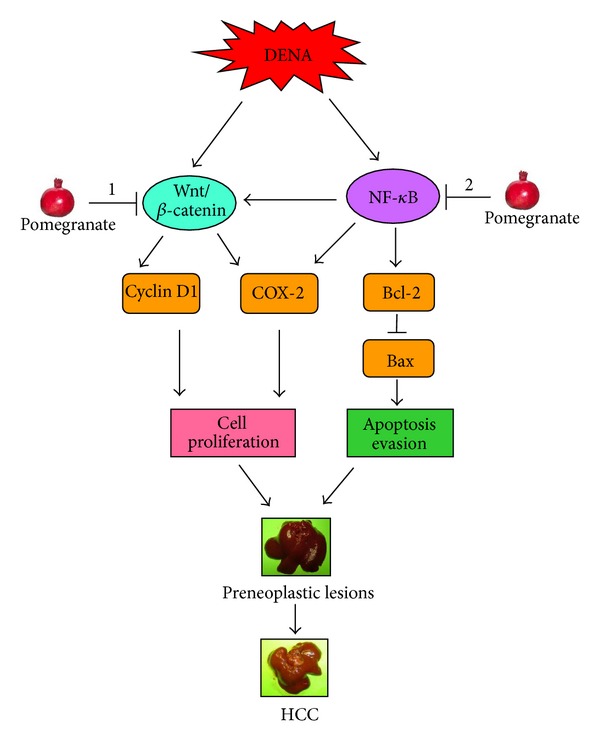
Schematic representation of the possible molecular mechanisms of pomegranate-mediated chemoprevention of experimental hepatocarcinogenesis in rats. COX-2: cyclooxygenase-2; DENA: diethylnitrosamine; HCC: hepatocellular carcinoma; NF-*κ*B: nuclear factor-*κ*B. DENA simultaneously activates Wnt/*β*-catenin and NF-*κ*B signaling pathways, which in turn, result in an elevated expression of target genes, including cyclin D1, COX-2, and Bcl-2 and reduced expression of Bax. All these features contribute to accelerated hepatocyte proliferation and evasion of apoptosis, which results in development of preneoplastic lesions and subsequently HCC. Pomegranate phytochemicals block interconnected Wnt/*β*-catenin and NF-*κ*B signaling pathways, which lead to antiproliferative and proapoptotic effects contributing to chemoprevention of HCC. ↓: Activation and ⊥: downregulation. 1 and 2 indicate results of this study and that from [35], respectively.

The results of our present study undoubtedly establish that pomegranate bioactive constituents suppress cell proliferation (PCNA), regulate cell cycle progression (cyclin D1), and induce programmed cell death (apoptosis) during DENA-initiated and PB-promoted hepatic tumorigenesis in Sprague-Dawley rats. Pomegranate-mediated apoptogenic signal during experimental hepatocarcinogenesis may be transmitted through up-relegation of proapoptotic protein Bax and downmodulation of antiapoptotic protein Bcl-2. We also present data that show, for the first time, that pomegranate phytochemicals diminish cell proliferation and survival possibly through blockade of activated canonical Wnt/*β*-catenin signaling in hepatocarcinogen-exposed animals. The interference of Wnt/*β*-catenin signaling may be achieved, at least in part, through degradation of *β*-catenin protein, the cardinal effector of Wnt pathway, by up-regulation of GSK-3*β*. This is further supported by the diminished hepatic expression of cyclin D1, a *β*-catenin-dependent target gene, and an indicator of activation of Wnt/*β*-catenin pathway. Thus, our study provides encouraging preclinical evidence and novel mechanism of targeting activated Wnt/*β*-catenin signaling by pomegranate products to achieve chemoprevention of hepatocellular cancer. Previously, we have shown modulation of the NF-*κ*B pathway by pomegranate constituents in DENA hepatocarcinogenesis in rats [35]. As functional cross regulation between the NF-*κ*B and Wnt/*β*-catenin signaling pathways has emerged as a pivotal mechanism for the regulation of a diverse array of genes involved in tumorigenesis, including HCC [37–40], results reported here in conjunction with our earlier communication [35] indisputably establish the “proof-of-principle” of simultaneously targeting two interconnected molecular circuits, namely, canonical NF-*κ*B and Wnt/*β*-catenin pathways, to achieve prevention of hepatocellular cancer (summarized in Figure 7). Obviously, these do not exclude the possibility of other mechanisms. Overall, our present results based on a clinically relevant model of liver cancer coupled with an excellent safety profile of pomegranate bioactive substances may facilitate the development of pomegranate-derived agents as complex, synergistic drug for the prevention and therapy of liver cancer, which represents a complex disease.
